# Microstructural Neuroimaging of Frailty in Cognitively Normal Older Adults

**DOI:** 10.1093/geroni/igaa057.571

**Published:** 2020-12-16

**Authors:** Qu Tian, Susan Resnick, Bennett Landman, Luigi Ferrucci

**Affiliations:** 1 National Institute on Aging, Bethesda, Maryland, United States; 2 Vanderbilt University, Nashville, Tennessee, United States

## Abstract

Physical frailty is an age-related clinical syndrome that is related to adverse health outcomes, including cognitive impairment and dementia. Recent studies have shown structural neuroimaging correlates with frailty. However, most existing evidence relies on brain volumetric measures. Whether brain microstructure is associated with frailty and its spatial distribution have not been explored. In the Baltimore Longitudinal Study of Aging, we identified 776 cognitively normal participants aged 50 and older who had concurrent data on frailty and brain microstructure by diffusion tensor imaging (DTI), including mean diffusivity (MD) of gray matter and fractional anisotropy (FA) of white matter. We first identified neuroimaging markers that were associated with frailty score (0-5) and further examined their relationships with frailty status (0: non-frail, 1-2: pre-frail, 3+: frail) using multivariate linear regression. Models were adjusted for age, sex, race, years of education, and Apolipoprotein E e4 carrier status. DTI-based neuroimaging markers that were associated with frailty status were localized in the supplementary motor area of the frontal lobe, several subcortical regions (putamen, caudate), and body and splenium of corpus callosum. This study demonstrates for the first time that microstructure of both gray and white matter differs by frailty levels in cognitively normal older adults. Brain areas were not widespread, but mostly localized in gray matter subcortical motor areas and white matter corpus callosum. Whether changes in brain microstructure precede future frailty development warrants further investigation.

